# Inhibition of Kidney Proximal Tubular Glucose Reabsorption Does Not Prevent against Diabetic Nephropathy in Type 1 Diabetic eNOS Knockout Mice

**DOI:** 10.1371/journal.pone.0108994

**Published:** 2014-11-04

**Authors:** Muralikrishna Gangadharan Komala, Simon Gross, Harshini Mudaliar, Chunling Huang, Katherine Pegg, Amanda Mather, Sylvie Shen, Carol A. Pollock, Usha Panchapakesan

**Affiliations:** Renal Research Lab, Kolling Institute of Medical Research, Sydney University, Royal North Shore Hospital, St Leonards, Australia; The University of Manchester, United Kingdom

## Abstract

**Background and Objective:**

Sodium glucose cotransporter 2 (SGLT2) is the main luminal glucose transporter in the kidney. SGLT2 inhibition results in glycosuria and improved glycaemic control. Drugs inhibiting this transporter have recently been approved for clinical use and have been suggested to have potential renoprotective benefits by limiting glycotoxicity in the proximal tubule. We aimed to determine the renoprotective benefits of empagliflozin, an SGLT2 inhibitor, independent of its glucose lowering effect.

**Research Design and Methods:**

We induced diabetes using a low dose streptozotocin protocol in 7–8 week old endothelial nitric oxide (eNOS) synthase knockout mice. We measured fasting blood glucose on a monthly basis, terminal urinary albumin/creatinine ratio. Renal histology was assessed for inflammatory and fibrotic changes. Renal cortical mRNA transcription of inflammatory and profibrotic cytokines, glucose transporters and protein expression of SGLT2 and GLUT1 were determined. Outcomes were compared to diabetic animals receiving the angiotensin receptor blocker telmisartan (current best practice).

**Results:**

Diabetic mice had high matched blood glucose levels. Empagliflozin did not attenuate diabetes-induced albuminuria, unlike telmisartan. Empagliflozin did not improve glomerulosclerosis, tubular atrophy, tubulointerstitial inflammation or fibrosis, while telmisartan attenuated these. Empagliflozin did not modify tubular toll-like receptor-2 expression in diabetic mice. Empagliflozin did not reduce the upregulation of macrophage chemoattractant protein-1 (MCP-1), transforming growth factor β1 and fibronectin mRNA observed in the diabetic animals, while telmisartan decreased transcription of MCP-1 and fibronectin. Empagliflozin increased GLUT1 mRNA expression and telmisartan increased SGLT2 mRNA expression in comparison to untreated diabetic mice. However no significant difference was found in protein expression of GLUT1 or SGLT2 among the different groups.

**Conclusion:**

Hence SGLT2 inhibition does not have renoprotective benefits independent of glucose lowering.

## Introduction

Diabetic nephropathy is the commonest cause of chronic kidney disease worldwide [1]. Current best practice in the management of diabetic nephropathy involves tight glycaemic and blood pressure control, which includes specific blockade of the renin, angiotensin aldosterone systems [2], [3]. Although treatment options for patients have expanded in recent years, this has not translated to a reduction in the incidence of diabetic nephropathy [4]. Hence there is a need for novel agents that confer renoprotection.

Sodium glucose cotransporter 2 inhibitors (SGLT2i) are novel diabetic agents that block glucose entry into the kidney proximal tubular cell (PTC), resulting in glycosuria and lowering of blood glucose levels and have the added advantage of not inducing weight gain or hypoglycaemia [5], [6].

SGLTs are located on the luminal aspect of the proximal tubule (PT) and able to transport sodium and glucose from the ultrafiltrate into the cell due to a sodium concentration gradient, generated by the basolateral Na, K-ATPase pump [7]. Sodium glucose cotransporter 2 (SGLT2) is the major luminal glucose transporter located in the S1 and S2 segments of the PT, whilst sodium glucose cotransporter 1 (SGLT1) in the S3 segment contributes to less than 10% of total luminal glucose transport [8]. On the basolateral side of the cell, glucose is then passively transported via facilitative glucose transporters (GLUTs) into the vasculature. In the early segments of the kidney PT, SGLT2 on the apical membrane is coupled with GLUT2 on the basolateral side and together they reabsorb upto 90% of filtered glucose under normoglycaemic conditions [8].

Hyperglycaemia induces activation of various pathways, which stimulates the production of proinflammatory and profibrotic cytokines relevant in diabetic nephropathy including TGFβ. The effects of high glucose are predominantly mediated through the hypertrophic and profibrotic cytokine, TGFβ which is overexpressed in diabetic nephropathy [9]. There is clear evidence of the damaging effects of TGFβ on PTC growth and function [10]–[12]. We and others have also shown evidence for TGFβ induced activation of the innate immunity pathway in diabetic nephropathy, in particular Toll like receptor 2 (TLR2) and its endogenous ligand High Mobility Group Box 1 (HMGB1) [13], [14]. We have previously defined the effects of high glucose in mediating inflammatory and profibrotic effects in the PTC [15], [16] and the specific effects of increased PTC sodium transport in early diabetes [17], [18]. Hence it is well established that high intracellular glucose alters intracellular metabolism and promotes inflammatory and profibrotic cytokines resulting in the development of diabetic nephropathy [12], [15], [19].

We have previously shown using human kidney PTC *in vitro* that empagliflozin, an SGLT2i (provided by Boehringer Ingelheim, Germany), was able to reduce high glucose induced tubular expression of inflammatory and fibrotic markers. In the short term, this occurred without a compensatory increase in SGLT1 or GLUT2 expression [20].

This provided proof of concept to extend these studies to a validated small animal model of diabetic nephropathy. An important aspect of our experimental design was to match glucose levels in all diabetic groups, so that any observed renal outcomes could be interpreted independent of the glucose lowering effect of empagliflozin, which has confounded the interpretation of previous studies to date. This was achieved by using a 5 day low dose protocol of intraperitoneal streptozotocin to induce diabetes [21] and long acting insulin in the diabetic mice to match glucose levels among all the experimental limbs.

## Materials and Methods

### Animal Model

Male *enos* knockout mice on a C57BL/6 background were purchased from Jackson laboratory, USA. Mice were housed singly in filter top cages in a pathogen free facility and had free access to standard chow and drinking water. Diabetes was induced by a low-dose streptozotocin (STZ) protocol. Mice received intraperitoneal injections of STZ (55 mg/kg daily for 5 days) at 7–8 weeks of age. Control mice received citrate buffer injections (pH 4.5). Blood glucose was tested using a glucometer (Accuchek Nano, Roche) two weeks after STZ through tail vein blood collection. Diabetes was defined by blood glucose greater than 16 mmol/L after a six-hour daytime fast. Mice with levels below 16 mmol/L were excluded from the study. Fasting blood glucose levels were measured monthly. Long acting insulin (Insulin Glargine, Sanofi Aventis, Australia) was initiated as required from 10 weeks of age and was administered thrice weekly if the blood sugar exceeded 28 mmol/L or if they had lost weight greater than 25% from baseline. The study was approved by the Royal North Shore Hospital Ethics Committee (protocol number 1101-003A). The Australian Code of Practice for the Care and Use of Animals for Scientific Purposes was followed in this study. Animals were anaesthetised using short inhalational anaesthesia with 2% isoflurane for minor procedures. Animals were euthanised under 2% isoflurane anaesthesia using cardiac puncture terminally.

### Experimental Design

The SGLT2i empagliflozin (provided by Boehringer-Ingelheim Germany) was administered by daily oral gavage (Instech Lab, USA) using 1% hydroxyethylcellulose (Sigma Aldrich) as a vehicle. Current best practice is renin-angiotensin-aldosterone blockade. Hence telmisartan (Boehringer-Ingelheim, Germany) at a dose of 3 mg/kg/day was administered in drinking water as a comparative limb. Empagliflozin and telmisartan were initiated at 13 weeks of age. Mice were killed at 32 weeks of age. The groups were as below.

1. Control (ctrl, n = 12)
2. Control receiving empagliflozin 10 mg/kg daily (ctrl+empa, n = 8)
3. Diabetic (dm, n = 12)
4. Diabetic receiving empagliflozin 10 mg/kg daily (dm+empa, n = 10)
5. Diabetic receiving telmisartan, 3 mg/kg in drinking water (dm+tel = 7)

### Measurement of Physiological Parameters

Body weight was assessed monthly. Blood pressure was measured using a noninvasive tail vein cuff method (CODA BP apparatus, Kent Scientific, USA) preterminally (Table 1).

**Table 1:** Physical and clinical parameters.

	Control (n = 12)	Control +empagliflozin (n = 8)	Diabetic (n = 12)	Diabetic +empagliflozin (n = 10)	Diabetic +telmisartan (n = 7)
Gain in weight (gram)	5.4±0.5	3.9±1.0	2.4±0.7**	0.8±0.6**	3.1±0.6*
Left kidney/body weight ratio (%)	0.77±0.04	0.80±0.03	0.91±0.06	0.82±0.08	0.89±0.08
Average blood sugar (mmol/L)	11.0±0.3	11.5±0.4	21.1±0.4**	22.1±0.6**	22.9±0.7**
Systolic BP (mm Hg)	120±2	128±4	117±2	122±6	118±10
24 hour urine glucose excretion (µmol/day)	2.6±0.3	456.5±74.4	3038.0±864.8*	3551.0±35.9**	3462.0±320.4**
Mid experimental albumin/creatinine ratio (µg/mg)	133±25	197±48	987±336*	463±136	180±121
Terminal albumin/creatinine ratio (µg/mg)	224±36	290±56	1474±388*	1697±596*	80±30#

Data are mean ± standard error of mean.

* = P<0.05 vs control group,

** = P<0.001 vs control group,

# = P<0.05 vs diabetic group.

### Urine Biochemistry

Urine was collected at three different time points (48 hour post initiation of treatment, 4–6 weeks after initiation of treatment using metabolic cages and terminally using bladder puncture). Urine creatinine was measured using a picric acid method (Creatinine Companion, Exocell Inc., USA). Urinary glucose was measured using Abbot Architect C16000 analyser. Urine albumin was measured using Elisa (Albuwel, Exocell Inc., USA).

### Kidney Tissue Harvest

The unperfused left kidney was harvested and snap frozen after embedding in OCT compound. The right kidney was perfused with phosphate buffered saline (PBS) followed by 4% paraformaldehyde (PFA) and subsequently fixed in 10% neutral buffered formalin for 24–48 hours.

### Histology

Formalin fixed paraffin embedded (FFPE) kidney sections were stained with Masson's Trichrome, Sirius Red and Periodic Acid Schiff. Assessment of histological change was done in a blinded manner. The Glomerulosclerotic index (GSI) was calculated based on previously described methodology [22]. Atrophic tubules were defined by dilatation, epithelial shedding and thinning of epithelium. Tubular damage was scored by counting the number of atrophic tubules per 400 tubules at X 200 magnification.

Immunohistochemistry for fibronectin and TLR2 were done on 4 micron paraffin embedded sections using rabbit anti mouse fibronectin (F3468, Sigma Aldrich, USA) at a concentration of 1∶2000 and rabbit anti mouse TLR2 (Imgenex, San Diego, California) at a concentration of 1∶250. The chromogenic reaction was carried out with 3, 3′-diaminobenzidine chromogen (Dako, Australia) solution for 10 minutes. Immunohistochemistry for F4/80 staining was done on 10 micron frozen section slides. They were incubated with rat anti mouse F4/80 (MCA497R, ABD Serotec, USA) at a concentration of 1∶100 for one hour followed by HRP tagged goat anti Rat antibody at a concentration of 1∶200 (ABD Serotec, USA).

F4/80 positive cells per high power field was counted and averaged for each slide. The degree of interstitial collagen content in Masson's and Sirius red stains were assessed in a blinded manner using Image J by identifying the percentage of interstitial collagen positive region at X 200 magnification in 5 randomly selected regions. Glomerular fibronectin was quantified using Image J at X 400 magnification after selecting 10–15 glomeruli at random and quantifying the percentage of fibronectin positive area in each glomerulus.

### Real Time PCR Experiments

Total RNA was extracted from kidney tissue using Trizol. cDNA was synthesised using Superscript III (Life Technologies) first strand synthesis. The cDNA was subjected to standard curve measurement to ensure efficiency prior to real time PCR, which was done using SYBR green (Life Technologies, Australia) for MCP-1, fibronectin, TGFβ, collagen IV, GLUT1 and GLUT2 using actin as the endogenous control. PCR for SGLT2 and SGLT1 were done using Taqman PCR Universal Mastermix (Applied Biosystems). The RTPCR was performed on the AB7900 machine (Applied Biosystems, Australia). Gene expression was quantified relative to actin. The primers are listed in Table 2.

**Table 2:** PCR primer sequences.

Target	Forward	Reverse	Source
MCP-1	GCCTGCTGTTCACAGTTGC	CAGGTGAGTGGGGCGTTA	Sigma
Collagen IV	TTAAAGGACTCCAGGGACCAC	CCCACTGAGCCTGTCACAC	Sigma
Fibronectin	CACGGAGGCCACCATTACT	CTTCAGGGCAATGACGTAGAT	Sigma
TGFβ	TCAGACATTCGGGAAGCAGT	ACGCCAGGAATTGTTGCTAT	Sigma
Actin	AAGGCCAAGCGTGAAAAGAT	GTGGTAGGACCAGAGGCATAC	Sigma
GLUT1	AACATGGAACCACCGCTACG	GTGGTGAGTGTGGTGGATGG	Sigma
GLUT2	ATCGCCCTCTGCTTCCAGTAC	GAACACGTAAGGCCCAAGGA	Sigma
SGLT1	Mm00451203_m1 (Applied Biosystems)		
SGLT2	Mm00453831_m1 (Applied Biosystems)		

### Western Blot

Frozen tissue was homogenized with Quiagen Tissue Ruptur in 1.5 ml of cold 20 mM HEPES buffer, pH 7.2, containing 1 mM EGTA, 210 mM mannitol, 70 mM sucrose and centrifuged at 1.500×g for 5 min at 4°C. Samples were then analysed by SDS gel electrophoresis (Novex, Life technologies, Australia) and electroblotted to Hybond Nitrocellulose membranes (Amersham Pharmacia Biotech, Bucks, UK). Membranes were then probed with primary antibodies to GLUT1 (ab652, Abcam, Cambridge, UK), SGLT2 (sc98975, Santa Cruz, USA) and actin (Santa Cruz, USA). Proteins were visualized using Luminata Western HRP Substrate (Millipore) in a LAS 4000 image reader (GE Healthcare Life Sciences). Analysis was performed using Image J software (NIH, USA).

### Statistical Analysis

Statistical analysis was done using Graph Prism 6. Data are expressed as mean ± standard error of mean. A P value <0.05 was considered statistically significant. Significance was assessed using paired t- test for analysing the difference in insulin requirement of diabetic mice before and after initiation of empagliflozin. Blood glucose profile during the study was measured using repeated measures Anova. Anova with Bonferroni's correction for multiple comparisons was used for all other statistical analysis.

## Results

### Diabetic Mice had Similar Fasting Glucose Levels

**Figure 1:**
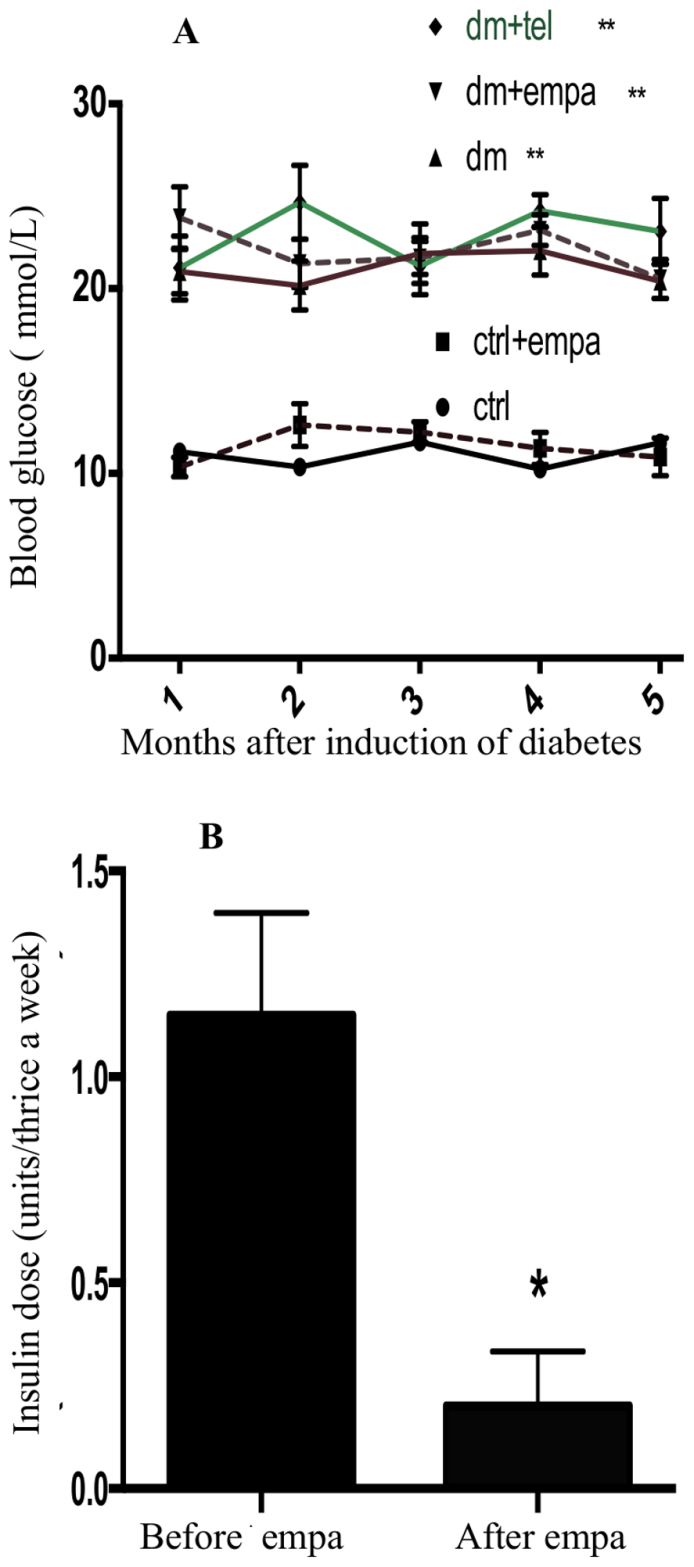
Blood glucose levels among diabetic mice were matched throughout the study and showed similar glucose levels among the diabetic mice at 1, 2, 3, 4 and 5 months post diabetes induction and were significantly higher than control mice (A). Empagliflozin reduced insulin requirement in diabetic mice (B). The comparison was made between average insulin requirement per dose administered thrice weekly, before and after initiation of empagliflozin. Data are expressed as mean ± SEM with * = P<0.05 and ** = P<0.001 vs ctrl.

The blood glucose levels were measured monthly, starting two weeks after induction of diabetes and average values were calculated over the duration of the experiment for all groups. The diabetic mice displayed significantly elevated blood glucose levels at 21.4 mmol/L after induction with streptozotocin (Table 1) maintained with long acting insulin and empagliflozin treatments, while the glucose level was matched in all the diabetic limbs throughout the experiment (Figure 1A) as planned.

### Empagliflozin Induced Glycosuria in Non Diabetic Mice

The control + empagliflozin group displayed nearly 200 fold increase in urinary glucose levels compared to the control mice (Table 1). The urinary glucose excretion among all diabetic mice was significantly higher than control mice (Table 1).

### Empagliflozin Reduced Insulin Requirement and Accentuated Poor Weight Gain in Diabetic Mice

Comparisons were made between the thrice weekly insulin dose of diabetic mice in the diabetic + empagliflozin group, before and after initiation of empagliflozin. The insulin requirements of the diabetic mice were significantly reduced after initiation of empagliflozin (Figure 1B). All diabetic mice had poor weight gain which was pronounced in the diabetic + empagliflozin group (Table 1).

### Telmisartan but Not Empagliflozin Reduced Terminal Urinary Albumin Excretion in Diabetic Mice

The terminal urinary albumin to creatinine ratio was elevated in the diabetic mice. Treatment with empagliflozin did not improve albuminuria in diabetic mice. This was significantly reduced by treatment with telmisartan in diabetic mice. In the early stages of the illness, SGLT2 inhibition shows a non significant trend towards improving albuminuria in diabetic mice, which is not maintained as the disease progresses (Table 1).

### Telmisartan but not empagliflozin reduced the degree of glomerulosclerosis and glomerular fibronectin deposition in diabetic mice

**Figure 2:**
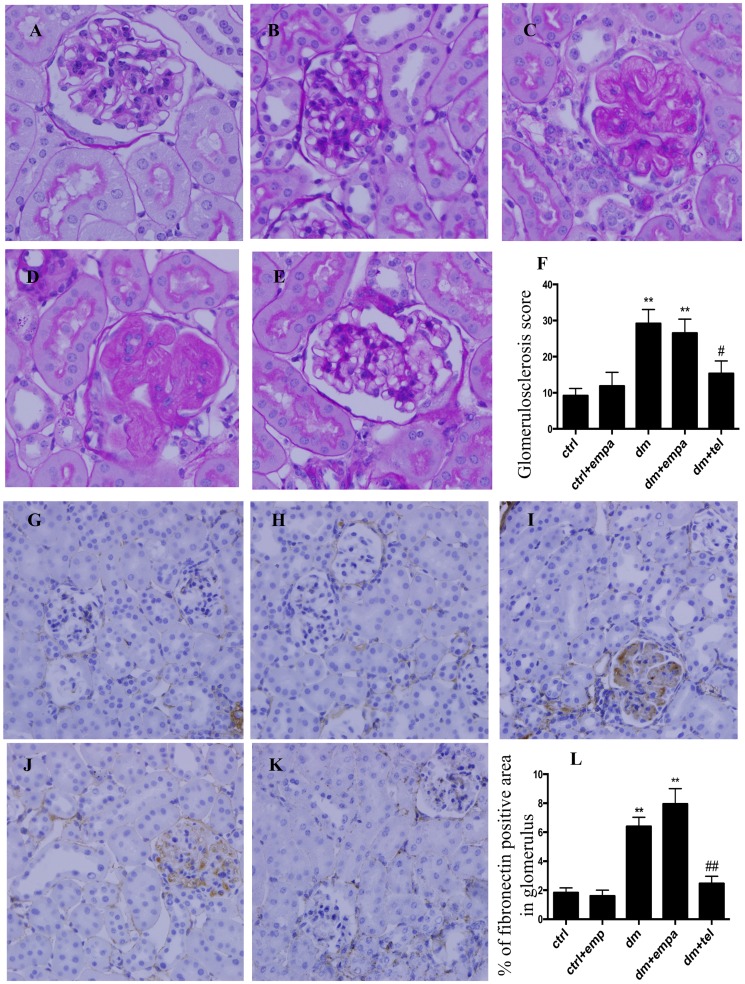
Diabetic mice demonstrated increased glomerulosclerosis and glomerular fibronectin deposition, which was improved by telmisartan but not by empagliflozin. Representative photographs of PAS stained sections for A) ctrl, B) ctrl + empa, C) dm, D) dm + empa and E) dm + tel groups and quantification of glomerulosclerosis by glomerulosclerotic index (F). Representative photographs of immunohistochemistry for glomerular fibronectin in G) ctrl, H) ctrl + empa, I) dm, J) dm + empa and K) dm + tel groups and quantification of glomerular fibronectin by Image J (L) (Magnification = original X400). Data are expressed as mean ± SEM with ** = P<0.001 vs ctrl, # = P<0.05 vs dm and ## = P<0.001 vs dm.

The untreated diabetic mice (Figure 2C) developed significant glomerulosclerosis in comparison with control mice (Figure 2A) Treatment with empagliflozin showed no improvement in the glomerulosclerotic index in diabetic mice (Figure 2D), whereas diabetics treated with telmisartan (Figure 2E) had significantly lower glomerulosclerotic scores. The diabetic mice (Figure 2I) developed significant glomerular fibronectin deposition in comparison with control mice (Figure 2G). There was no improvement with concurrent empagliflozin therapy (Figure 2J), while telmisartan was associated with a significant reduction in glomerular fibronectin deposition in diabetic mice (Figure 2K).

### Empagliflozin did not reduce tubulointerstitial inflammation in diabetic mice

**Figure 3:**
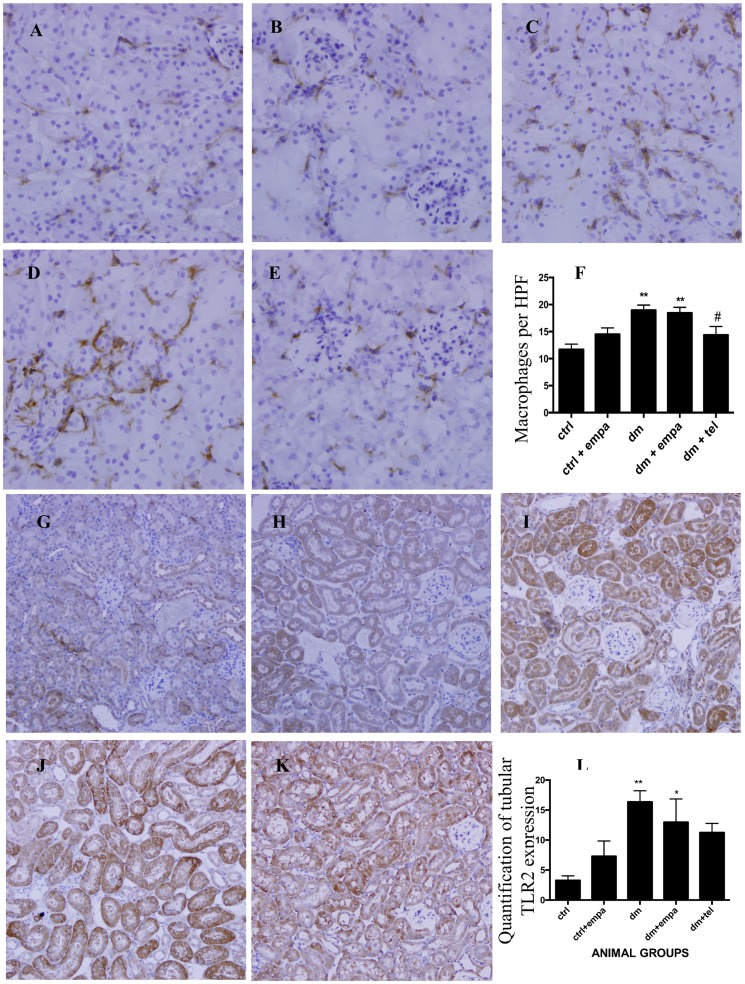
Diabetic animals demonstrated increased tubulointerstitial inflammation, which was not ameliorated by empagliflozin. Representative photographs of immunohistochemistry for tubulointerstitial F4/80 stain for activated macrophages in A) ctrl, B) ctrl + empa, C) dm, D) dm + empa, E) dm + tel groups (Magnification  = original X 400) and quantification of F4/80 positive cells in tubulointerstitium (F) which was done by calculating the average number of F4/80 positive cells per high power field (HPF) in each group. Representative photographs of immunohistochemistry for tubular TLR2 in G) ctrl, H) ctrl + empa, I) dm, J) dm + empa, K) dm + tel groups (Magnification  = original X 200) and quantification of tubular TLR2 expression by Image J (L). (Data are expressed as mean ± SEM with * = P<0.05 vs ctrl, ** = P<0.001 vs ctrl and # = P<0.05 vs dm).

The diabetic mice (Figure 3C) developed significantly increased tubulointerstitial infiltration of activated macrophages, demonstrated by F4/80 staining, compared to controls (Figure 3A). Concurrent treatment with empagliflozin did not improve this in diabetic mice (Figure 3D). However, telmisartan therapy significantly reduced macrophage infiltration in diabetic (Figure 3E). The diabetic mice also displayed increased tubular expression of TLR2 (Figure 3I). Empagliflozin did not modify the upregulation of TLR2 while telmisartan treated diabetic mice showed TLR2 expression not significantly different to that in control mice (Figure 3J and K).

### Telmisartan but not empagliflozin reduced the degree of tubular atrophy and tubulointerstitial fibrosis in diabetic mice

**Figure 4:**
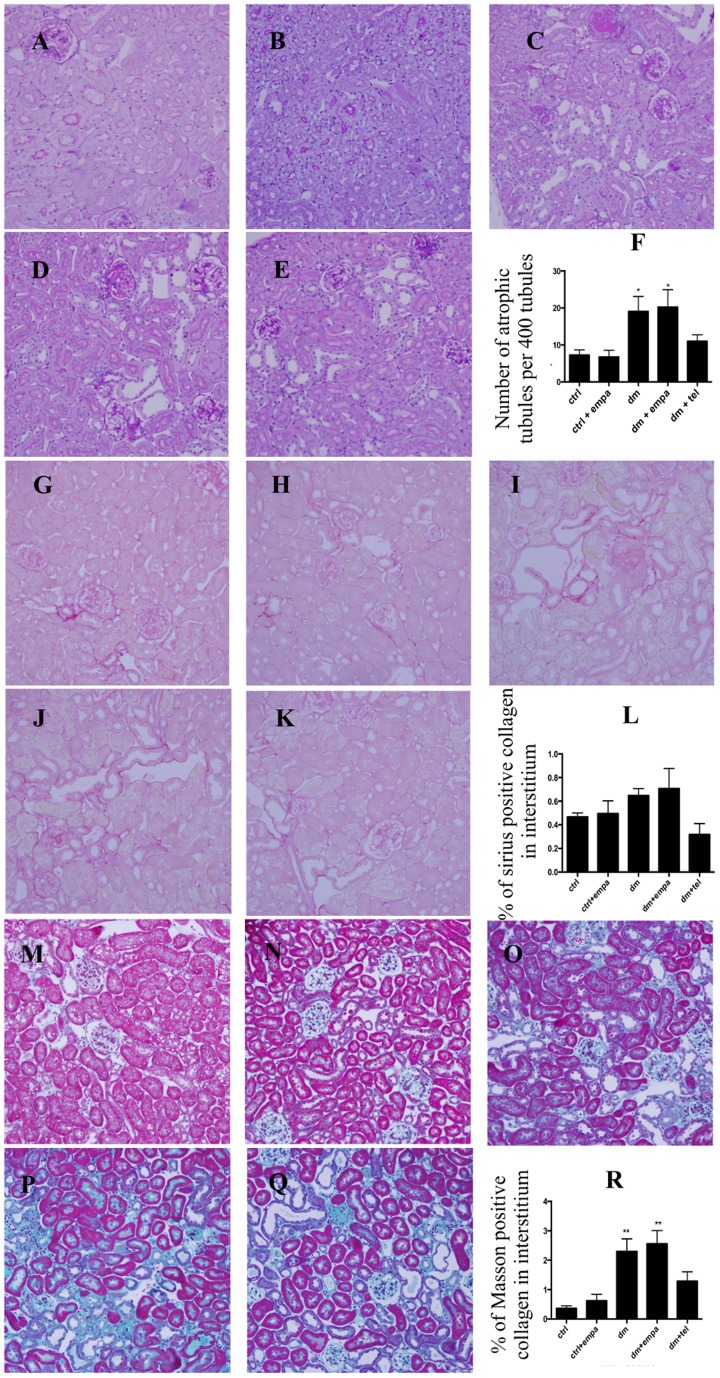
Diabetic mice demonstrated tubular atrophy and tubulointerstitial fibrosis, which was not improved by empagliflozin and partially reduced by telmisartan. Representative photographs of PAS stained sections of tubulointerstitium in A) ctrl, B) ctrl + empa, C) dm, D) dm + empa, E) dm + tel groups and F) Quantification of tubular atrophy in all groups was done by counting the number of atrophic tubules per 400 tubule count (Data are expressed as mean ± SEM with * = P<0.05 vs ctrl). Representative photographs of tubulointerstitial picrosirius red stain in G) ctrl, H) ctrl + empa, I) dm, J) dm + empa, K) dm + tel groups and L) Quantification of tubulointerstitial Sirius red positive collagen content by Image J. Representative photographs of Masson's stain in M) ctrl, N) ctrl + empa, O) dm, P) dm + empa, Q) dm + tel groups and R) Quantification of Masson's positive collagen content by Image J (Magnification  = original X 200). (Data are expressed as mean ± SEM with * = P<0.05 vs ctrl, ** = P<0.001 vs ctrl).

The diabetic mice (Figure 4C) developed significant tubular atrophy in comparison with control mice (Figure 4A). Empagliflozin did not reduce tubular atrophy (Figure 4D), while temisartan treated diabetic mice showed tubular atrophy not significantly different to control mice (Figure 4E). The degree of interstitial fibrosis was assessed by Sirius Red sensitive collagen staining (Figures 4G-K) and Masson's staining (Figures 4M-Q). The diabetic mice showed a non significant trend towards increased collagen deposition with the sirius red stain. However with the masson's stain, the diabetic mice had significantly increased collagen deposition (Figure 4O), which was not improved by empagliflozin (Figure 4P). Telmisartan treated diabetic mice showed tubulointerstitial fibrosis not significantly different to that of control mice (Figure 4Q).

### Empagliflozin did not improve the renal cortical transcription of inflammatory or profibrotic cytokines in diabetic mice

**Figure 5:**
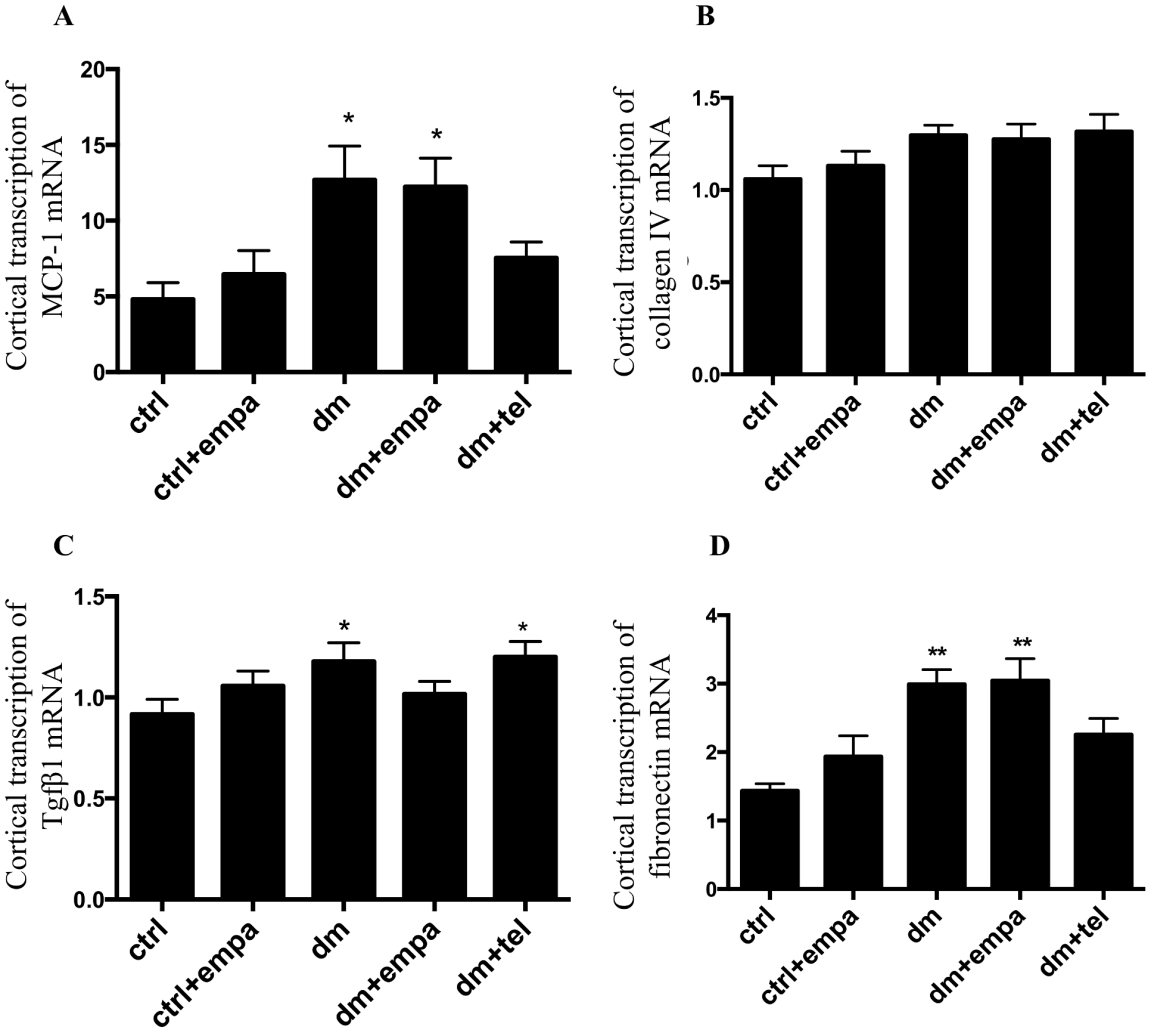
Diabetic mice showed increased renal cortical transcription of inflammatory and fibrotic cytokines, which was not improved by empagliflozin. Real time PCR results for Renal cortical transcription of A) MCP-1, B) Collagen 4, C) TGFβ and D) Fibronectin relative to actin (Data are expressed as mean ± SEM with * = P<0.05 vs ctrl and ** = P<0.001 vs ctrl).

To determine whether empagliflozin or telmisartan modulated the cortical transcription of MCP-1, collagen IV, fibronectin and TGFβ in diabetes, we performed real time PCR from RNA derived from renal cortical tissue. The transcription of MCP-1, fibronectin and TGFβ was increased in diabetic mice and was unchanged by empagliflozin (Figure 5). Diabetic mice treated with telmisartan displayed MCP-1 (Figure 5A) and fibronectin (Figure 5D) transcription, which was not significantly different to control mice although TGFβ transcription was noted to be increased in this group (Figure 5C). No change was noted in the transcription of collagen IV between control and diabetic mice (Figure 5B).

### Empagliflozin significantly increased GLUT1 transcription in diabetic mice although no difference was noted in GLUT1 protein expression

**Figure 6:**
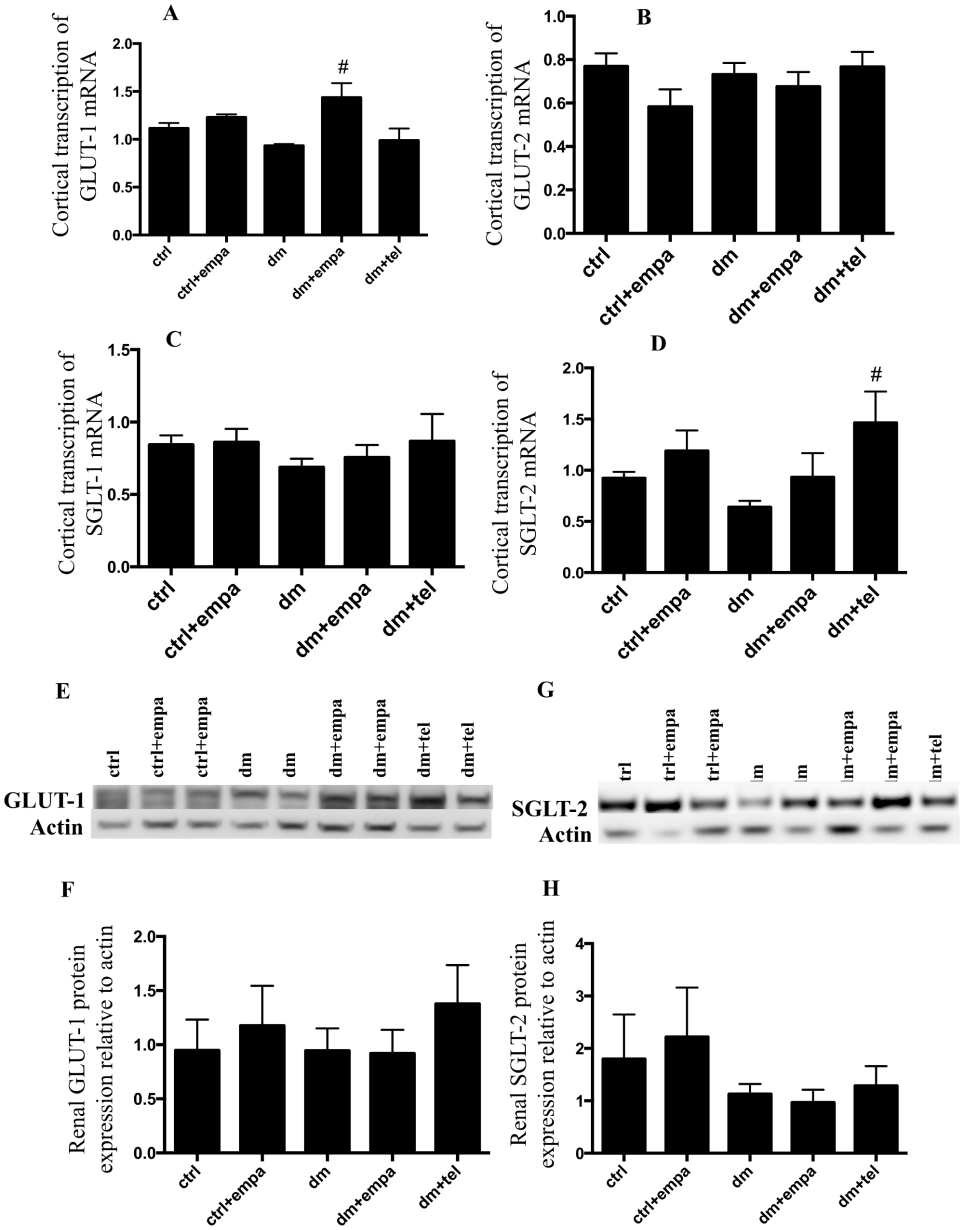
Empagliflozin increased renal GLUT1 transcription in comparison with untreated diabetic mice although renal protein expression of GLUT1 and SGLT2 were unchanged. Real time PCR results for A) GLUT1, B) GLUT2, C) SGLT1 and D) SGLT2 expressed relative to actin. Expression levels of renal GLUT1 protein (E and F) and SGLT2 (G and H) relative to actin (Data are expressed as mean ± SEM with # = P<0.05 vs dm).

There was no difference in mRNA transcription of any glucose transporters between diabetic and non diabetic mice (Figure 6). Empagliflozin treated diabetic mice showed a significant increase in GLUT1 transcription compared to other diabetic mice (Figure 6A) and telmisartan treated diabetic mice showed increased SGLT2 transcription compared to diabetic mice (Figure 6D). However there was no difference in either SGLT2 or GLUT1 protein expression among the different groups (Figure 6E-H).

## Discussion

To our knowledge this is the first study to evaluate the renoprotective effects of SGLT2 inhibition independent of glucose lowering. We used a type 1 diabetic enos knockout mouse model, validated by the Animal Models of Diabetic Complications Consortium (AMDCC) [21], with matched glycaemia across experimental groups. Although we have previously demonstrated that empagliflozin reduced high glucose induced expression of inflammatory and fibrotic markers in *in vitro* studies of human kidney PTC, our results show that it did not confer renoprotection in this *in vivo* model. This was in contrast to the renoprotection afforded by the angiotensin receptor blocker (ARB) telmisartan.

The strength of our study is based on the determination of renal parameters in a setting of matched high blood glucose levels among diabetic groups. In our study, empagliflozin caused a 200 fold increase in glycosuria in control mice compared to control mice not receiving the drug, which confirms that empagliflozin was absorbed and achieved sufficient concentration in the lumen to actively inhibit PTC SGLT2. Furthermore the diabetic mice receiving empagliflozin had less insulin requirements and less weight gain reflecting the efficacy of empagliflozin in inducing glycosuria in our model. As the effectiveness of SGLT2 inhibition is dependent on the creatinine clearance with decreased efficiency as creatinine clearance decreases we would expect the efficacy to decrease as the severity of diabetic nephropathy increases [23].

In contrast to our findings, there are recent animal studies wherein SGLT2 inhibitors have shown significant renal benefit [24], [25]. There are however important differences in these studies from our current study. In the study by Nagata et al, a type 2 diabetic model was used and diabetic mice treated with the SGLT2i tofogliflozin had lower plasma glucose levels, making interpretation of renal benefit independent of glucose lowering difficult. The second study by Kojima et al utilized older (> I year old) type 2 diabetic rats with established diabetic nephropathy and showed that although matched for glucose levels the diabetic mice receiving the SGLT2i luseogliflozin had better renal outcomes than the mice receiving insulin. The mice receiving insulin had 10 fold higher plasma insulin levels. Studies have shown that insulin itself can increase TGFβ-1 gene expression by mesangial cells [26] and can promote glomerular and interstitial fibrosis [27]. Furthermore, in a recent publication, Wright et al has described the probable role of insulin as an agonist in stimulating SGLT2 activity through activation of protein kinase A and C [28]. This would explain an increase in glucose reabsorption in type 2 diabetic mice with high insulin levels and provide an explanation for better outcomes with SGLT2 inhibition in this model. Current data available from the registration trials of SGLT2 inhibitors is limited, but suggests a stable reduction in estimated GFR than reverts to baseline after cessation of the drug. This occurs in association with a reduction in albuminuria. However, renoprotective benefits cannot be concluded from available clinical data.

Our studies are consistent with two recent studies by Vallon et al. The first study used SGLT2 knockout mice showing that although there was an improvement in glucose control and glomerular hyperfiltration, there was no improvement in kidney growth or injury [29]. An important difference between our own study and this study is that there was an improvement in blood glucose among mice treated with empagliflozin. Moreover it was noted that after 18 weeks of diabetes, no increase in TGFβ expression was observed in the renal cortex by their group. Conversely, we did demonstrate increased transcription of TGFβ in the renal cortex of diabetic animals although there was no statistically significant improvement with empagliflozin. Similar to Vallon's study, we found a statistically non significant trend towards decreased SGLT2 protein expression in diabetic mice, which may have contributed to reduced renoprotection by empagliflozin. However this cannot explain the significant diabetic changes in empagliflozin treated mice in contrast to improvement in the telmisartan group. The second study by Vallon et al in Akita mice, a type 1 model of diabetes, demonstrated that empagliflozin was effective in reducing GFR independent of blood glucose but the reduction in albuminuria, kidney growth and inflammation was thought to be the result of concomitant glucose lowering [30]. Our study in contrast was designed to evaluate renal outcome independent of glucose levels, which has been a confounding variable in all studies to date.

There are several possible reasons for our findings. Firstly, as the blood glucose levels were similarly elevated among all diabetic mice, this has resulted in glomerular injury, as SGLT2 inhibition does not prevent glucose entry into the glomerular compartment. It is known that progressive glomerular injury can cause tubulointerstitial damage through a number of different mechanisms including misdirected filtration (filtrate leakage external to the tubular lumen), obstructed filtration and proteinuria as a result of damage to the glomerular filtration barrier [31]. This then initiates a cycle of progressive tubulointerstitial injury, which is not prevented by blocking glucose entry into the PTC. Secondly, the lack of renoprotection seen in our study could be the result of incomplete inhibition of glucose reabsorption by empagliflozin. We know that SGLT2 inhibition only prevents reabsorption of 40% of glucose and this could be due to multiple reasons [32]. GLUT1 is a major glucose transporter in mesangial cells and is a facilitative transporter of glucose in the S3 segment of the basolateral aspect of the tubular cell with bidirectional transport properties [33]. It is possible that hyperglycaemia could influence the movement of glucose into the tubular cell from the basolateral aspect and is not modified by empagliflozin. This is not dependent on an increase in GLUT1 protein expression.

The diabetic mice in our model had matched high glucose levels, which is not reflective of the well controlled diabetic patient. However this was necessary in the design of the experiment to test the hypothesis whether SGLT2 inhibition can offer renoprotection independent of glucose lowering. There is evidence to suggest that SGLT2i can reduce diabetic nephropathy when glycaemic control is optimal in a Type 2 diabetic animal model and is even more pronounced when used in combination with an angiotensin receptor blocker [25]. However this could have been due to glucose lowering rather than specific SGLT2 inhibition. In summary, we have shown that although empagliflozin reduces high glucose induced inflammatory and fibrotic markers in vitro; it does not have renoprotective benefits independent of glucose lowering in vivo.

## Supporting Information

Data S1 **Analysis results for physical parameters, histology and PCR.**
(XLSX)
